# Correlation of NHR-48 Transcriptional Modulator Expression with Selected CYP Genes’ Expression during Thiabendazole Treatment of *Anisakis simplex* (s.l.)?—An In Vitro Study

**DOI:** 10.3390/pathogens9121030

**Published:** 2020-12-09

**Authors:** Elżbieta Łopieńska-Biernat, Robert Stryiński, Łukasz Paukszto, Jan P. Jastrzębski

**Affiliations:** 1 Department of Biochemistry, Faculty of Biology and Biotechnology, University of Warmia and Mazury in Olsztyn, 10-719 Olsztyn, Poland; robert.stryinski@uwm.edu.pl; 2 Bioinformatics Core Facility, Faculty of Biology and Biotechnology, University of Warmia and Mazury in Olsztyn, Oczapowskiego 1A, 10-719 Olsztyn, Poland; lukasz.paukszto@uwm.edu.pl (Ł.P.); bioinformatyka@gmail.com (J.P.J.); 3 Department of Plant Physiology, Genetics and Biotechnology, Faculty of Biology and Biotechnology, University of Warmia and Mazury in Olsztyn, 10-719 Olsztyn, Poland

**Keywords:** *Anisakis simplex*, thiabendazole, cytochrome P450, nuclear hormone receptor

## Abstract

*Anisakis simplex* (s.l.) is a complex of three sibling (biological) species of parasitic nematodes of marine mammals, including *A. berlandi*, *A. pegreffii* and *A. simplex* (s.s.). It is characterized by a complex life cycle in which humans can become accidental hosts by consuming dishes made of raw or undercooked fish containing L3 larvae, which in many regions of the world is related to the national or regional culinary tradition. This has spurred scientific efforts to develop new methods for treating the disease, called anisakiasis, and to neutralize invasive L3. Thiabendazole (TBZ) is a wide-spectrum anthelminthic with a higher efficacy than albendazole, a drug whose long-term use induces resistance in many parasitic species. Cytochromes P450 participate in TBZ metabolism, and the expression of their genes is controlled by nuclear hormone receptors (NHR). This study aimed to examine the effects of TBZ on the above-described pathway in invasive larvae of *A. simplex* (s.l.). The efficacy of TBZ against *A. simplex* (s.l.) larvae was observed for the first time. Larvae were cultured in vitro for 72 h in a medium containing TBZ at five concentrations from 0.5 to 1.5 mM. However, the survival curves did not significantly differ from each other. This means that all of the concentrations of TBZ had a similar effect on the *A. simplex* (s.l.) L3 larvae during in vitro culture. Nevertheless, TBZ modified the expression of *nhr-48*, *cyp13a3* and *cyp1a1* genes in the L3 of *A. simplex* (s.l.).

## 1. Introduction

The *Anisakis simplex* (s.l.) is a complex of three sibling (biological) species of parasitic nematodes of marine mammals, including *A. berlandi*, *A. pegreffii* and *A. simplex* (s.s.) [1]. Adult parasites live in the stomach of fish-eating marine mammals, such as orcas, dolphins, seals and porpoises [2]. *A. simplex* (s.l.) has a complex life cycle in which humans can become accidental hosts [3]. Parasitic larvae produce proteolytic enzymes [4], penetrate gastrointestinal mucosa and cause mucosal inflammations known as anisakiasis [5]. Infections caused by *A. simplex* (s.l.), especially by *A. pegreffii*, and *A. simplex* (s.s.), produce gastrointestinal symptoms and are often accompanied by mild allergic reactions [1,6]. However, sudden and severe allergic reactions have been noted without gastric symptoms [7]. In several documented cases, allergic reactions to L3 of *A. simplex* (s.l.) have been reported in humans after the consumption of fish processed at high or low temperatures [8]. The above can be attributed to the thermal resistance of L3 stage proteins, whose allergenic properties are not eliminated under exposure to extreme temperatures [9].

The growing popularity of raw fish dishes has contributed to an increase in the incidence of anisakiasis, which has spurred scientific efforts to develop new methods for diagnosing and treating the disease and neutralizing invasive L3 larvae [5,10]. Anisakiasis is also treated pharmacologically. The efficacy of several anthelminthic drugs against *Anisakis* spp. has been tested, including ivermectin, albendazole and its derivatives [11,12,13,14,15]. Researchers have also tested the action of various natural substances against Anisakis larvae in vitro and in vivo, such as monoterpenes, geraniol, citronellal and *Matricaria chamomilla* oils [15,16,17,18]. These efforts were limited to the determination of the minimal effective dose and the onset of action of the tested drugs, rather than their mechanism of action.

Studies of *Caenorhabditis elegans* demonstrated that thiabendazole (TBZ); [4-(1H-1,3-benzodiazol-2-yl)-1,3-thiazole], a benzimidazole group drug, is one of the most effective anthelminthics [19]. The mechanism of action of TBZ relies on the polymerization of β-tubulin and the disruption of processes required for the formation of microtubules in cells. Some authors also suggest the inhibition of fumarate reductase, an enzyme that participates in the reductive citric acid cycle in parasite cells [20,21]. Regarding a possible mechanism, current evidence supports that benzimidazoles inhibit the microtubule-mediated apical vesicle transport of hydrolytic enzymes (digestive enzymes) in intestinal cells, which then become dispersed in the cytoplasm, where the intracytoplasmic digestion of intestinal cells/tissue might occur [22]. For all these reasons, TBZ is a highly effective anthelminthic drug. However, anthelmintics resistance is a serious worldwide problem. Gene mutations (single nucleotide polymorphism) reported between β-tubulin codons 198 and 200 also increase parasites’ resistance to TBZ [23]. Other findings suggest that an increased drug metabolism may play a role in the resistance to drugs from the benzimidazoles group. Studies of the resistant strain of *Haemonchus contortus* confirmed that this nematode was able to form many more metabolites from all the tested benzimidazoles than individuals of the sensitive strain [24]. Therefore, it seems urgent to describe the TBZ metabolism pathway due to its not-yet-high resistance in parasitic nematodes.

In mammals, drugs and other xenobiotics are mainly biotransformed by enzymes in endoplasmic reticulum membranes in hepatocytes. Endogenous and exogenous compounds are metabolized in two stages, I and II. Some drugs are transformed during a single phase, but in most cases the metabolites produced in phase I are transformed in phase II. Phase I involves oxidation, reduction and hydrolysis processes, which transform drugs into more polar compounds, whereas in phase II, drugs are fused with endogenous compounds, such as glucuronic acid, to produce mostly inactive substances that are excreted with urine or bile. There is evidence that in nematodes the major mode of benzimidazoles metabolism is glucosidation [25], which is extremely rare in mammals, and there are no reports of TBZ glucosidation in mammalian systems. This may represent an important difference between mammalian and nematode xenobiotic responses in general and could potentially be a target for future chemotherapeutics. Nevertheless, in mammals the TBZ is metabolized via hydroxylation at position 5 of the benzimidazole ring into 5-hydroxythiabendazole, which is then broken down into glucuronide and sulphate conjugates [26,27]. The purpose of both phases is to make drugs soluble in water and facilitate their excretion.

Phase I reactions involve, among others, cytochrome P450 enzymes (CYPs) [28]. Approximately 40% of the 5434 candidate genes of benzimidazole resistance across all identified quantitative trait loci in *C. elegans* have orthologs in clade III [29,30], which is where *A. simplex* (s.l.) belongs. It is reasonable to suppose that genes validated in this system as modulators of benzimidazole response also have counterparts in parasitic nematodes genomes. Studies of *C. elegans* demonstrated that CYPs participated in TBZ metabolism and that the expression of their genes was regulated by nuclear hormone receptors (NHR) [19,31]. CYPs are proteins that participate in oxidative phosphorylation in phase I of cell metabolism. Their expression is regulated by NHRs by binding specific ligands [32]. The regulation of drug detoxification genes in mammals occurs mainly by transcriptional events through the activation of specific nuclear receptors named “xenosensors”, but very little information exists on the mechanisms by which these genes are regulated in parasitic nematodes [33].

Due to this, the aim of this study was to determine the survival rate of *A. simplex* (s.l.) parasites exposed to TBZ and to describe the correlation of NHR-48 expression (transcriptional modulator) with selected CYP gene expression during the TBZ treatment of *A. simplex* (s.l.).

## 2. Results

### 2.1. Survival of Larvae after TBZ Exposure

**Figure 1:**
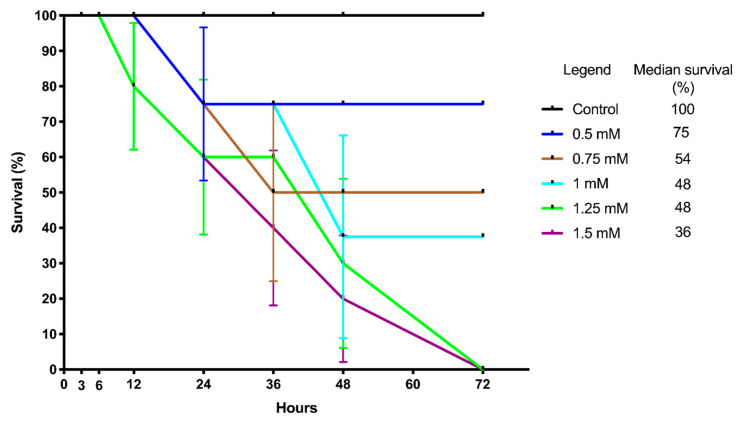
The Kaplan–Meier survival curve of *Anisakis simplex* (s.l.) L3 larvae exposed to different TBZ concentrations (0.5, 0.75, 1.0, 1.25 and 1.5 mM) in an in vitro culture for 72 h. The fraction survival errors bars were expressed as SE. Median survival was calculated and shown in %.

The survival curves were plotted to visualize the survival rate (%) of *A. simplex* (s.l.) L3 larvae after exposure to TBZ (Figure 1). The Log-rank test for the trend showed significant results (*p*-value = 0.0041; χ^2^ = 8.26; df = 1), which indicates that TBZ decreased the mobility and survival of *A. simplex* (s.l.) larvae cultured in vitro in all tested concentrations of TBZ when compared to the control. However, the Log-rank (Mentel–Cox) test showed (*p*-value = 0.1231; χ^2^ = 8.67; df = 5) that the survival curves did not significantly differ from each other. This means that all of the concentrations of TBZ have a similar effect on the *A. simplex* (s.l.) L3 larvae during the in vitro culture. Nevertheless, the lowest median survival (36%) was noted for the larvae cultured with 1.5 mM TBZ.

### 2.2. Bioinformatical Analyses

**Figure 2:**
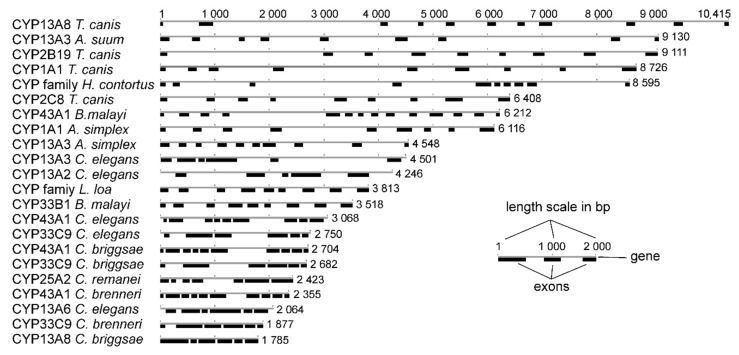
The exon/intron structure of CYP genes. *Anisakis simplex* (s.l.) sequences were compared to sequences of CYP genes of *Caenorhabditis* spp. and other chosen parasitic nematode species. See legend in the figure.

In the *A. simplex* (s.l.) genome, two CYP genes were found. The exon-intron structure of *cyp1a1* and *cyp13a3* was compared to other selected CYP genes (Figure 2). All of the analyzed genes showed a similar number of exons, ranging from five in *cyp13a3* in *C. elegans* to 14 in *cyp43a1* in *Brugia malayi*. The analyzed *A. simplex* (s.l.) genes showed nine and 10 exons in *cyp1a1* and *cyp13a3*, respectively. In all analyzed nematode species, the numbers of exons remained identical, whereas the lengths of exons were different.

**Figure 3:**
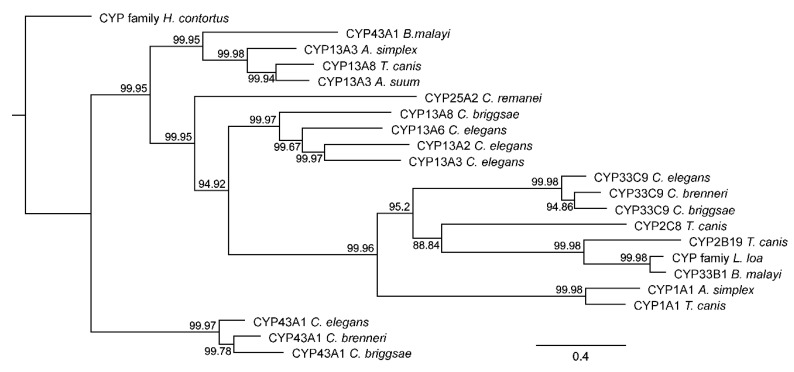
The phylogenetic tree of CYPs. Protein sequences of *Anisakis simplex* (s.l.), *Caenorhabditis* spp. and other chosen parasitic nematode species were compared. The clustering method used during the analysis was the neighbor joining method. Numbers in the figure show the phylogenetic similarity between the sequences (%).

The phylogenetic tree of CYPs (Figure 3) shows that branches on the tree correspond to individual CYP families, i.e., CYP13, CYP33 or CYP43. Interestingly, there were no parasitic and free-living nematodes on the same branch. It was found that the CYP 1 family is phylogenetically similar to the CYP 2 and CYP 33 families, and genes from the CYP 13 family showed a similarity to the genes from CYP 43 and CYP 25, although parasitic nematodes were clearly distinguished from free-living nematodes (Supplementary File 1).

**Figure 4:**
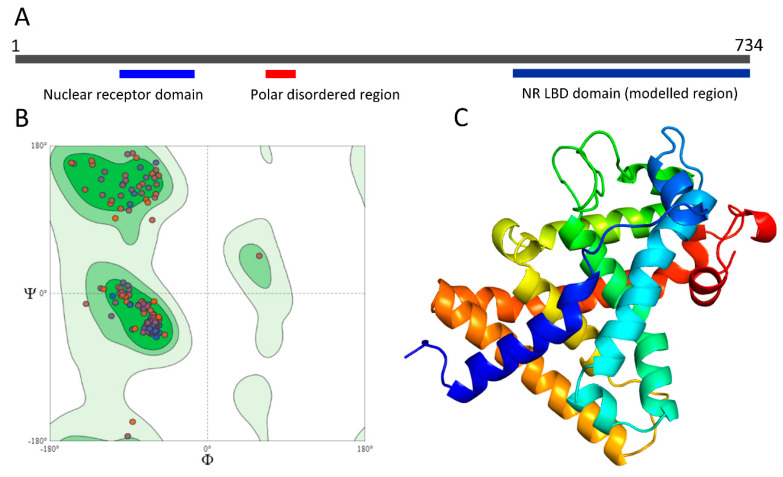
The structure of NHR-48. (**A**) A 734 amino acid-long sequence contains two nuclear receptor domains (blue bars) and one polar disordered region (red bar). (**B**) The Ramachandran plot of the modelled region (491–732) shows 98.33% favored residues and 0.83% outliers (the two residues: 652 ASN and 519 PRO). (**C**) Predicted 3D structure of the NR-LBD domain colored from the N′ end (blue) to C′ end (red).

The phylogenetic analysis of NHR showed that the *A. simplex* (s.l.) *nhr-48* gene was highly similar (82%) to *daf-12* of *Onchocerca volvulus* and *nhr-48* of *B. malayi*. Additionally, the *H. contortus daf-12* gene showed a strong relationship with *C. japonica nhr-8* and *daf-12* of *C. elegans* and *C. briggsae*. This showed a different situation than in CYPs’ phylogeny because NHRs showed a similarity between parasitic and free-living nematodes (Supplementary File 2). Moreover, the structure of NHR-48 (organism: *A. simplex*—UniProtKB: A0A0N9E5R4) was predicted (Figure 4). A 734 amino acid-long sequence (with a molecular weight of 80.26 kDa with a theoretical isoelectric point of 8.44) contains two domains (blue bars): the nuclear receptor domain (104–179 aa, InterPro annotation: PROSITE: PS51030) and ligand-binding domain (NR-LBD) domain (497–734 aa, InterPro annotation: PROSITE: PS51843), as well as one polar disordered region (red bar) (Figure 4A). A Ramachandran plot (Figure 4B) of the modelled region (491–732) showed 98.33% favored residues and 0.83% outliers (the two residues: 652 ASN and 519 PRO). A C-terminal NR-LBD, which plays a crucial role in ligand-mediated NHR activity, was modelled, and its 3D structure was shown (Figure 4C). The most accurate predicted model of NR-LBD has QMEAN = –1.73. The overall structure of NR-LBD is composed of 13 alpha-helices that are arranged into a three-layer antiparallel alpha-helical sandwich. A database search revealed the structure annotations and membership in the protein family and domain: InterPro—IPR035500, IPR000536, IPR001723, IPR001628, IPR013088; PFAM—PF00104, PF00105; PRINTS—PR00047; SMART—SM00399; PROSITE—PS51843, PS00031, PS51030.

### 2.3. qReal-Time PCR

**Figure 5:**
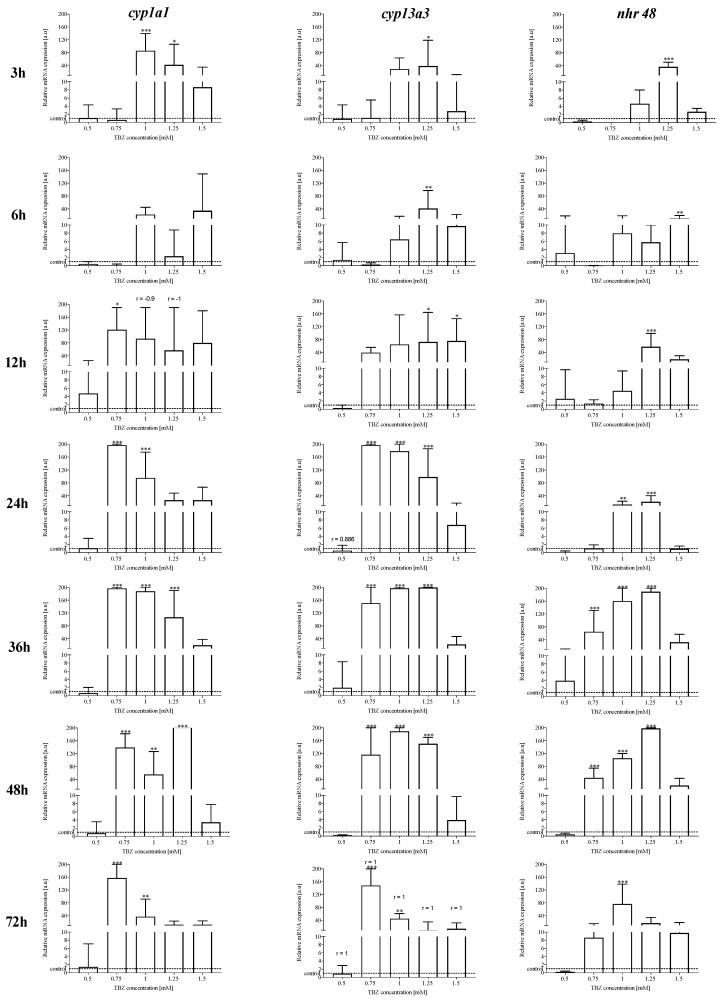
The expression of *nhr-48*, *cyp1a1* and *cyp13a3* in *Anisakis simplex* (s.l.) L3 larvae exposed to different TBZ concentrations (0.5, 0.75, 1.0, 1.25 and 1.5 mM) in an in vitro culture for 72 h. The depicted values are means of three replicates ± SD. The data were presented as the fold change in gene expression normalized to an endogenous reference gene and relative to the untreated control (relative quantification RQ = 1). Differences between means were assessed by Dunnett’s multiple comparisons test. *p*-values below 0.1234 (nonsignificant) were considered statistically significant, where 0.0332 (*), 0.0021 (**), <0.0001 (***). The Spearman correlation between the expression of *nhr-48* and *cyp1a1* or *cyp13a3* was marked with r above bars with correlated conditions only. The results were considered statistically significant when *p* ≤ 0.05 (one-tailed).

The TBZ influenced the expression of the *nhr-48*, *cyp13a3* and *cyp1a1* genes in most of the studied TBZ concentrations and times of exposure in L3 of *A. simplex* (s.l.) (Figure 5). The Spearman correlation test (Supplementary File 3) showed a strong negative correlation between *nhr-48* expression and *cyp1a1* expression at 12 h of exposure to TBZ in concentrations of 1 mM (r = −0.9) and 1.5 mM (r = −1). A strong positive correlation was noted at 24 h of the culture for 0.5 mM TBZ (r = 0.886) and at 72 h of the culture for all tested concentrations of TBZ (r = 1), where the increase in the expression of the *nhr-48* gene was proportional to the increase in the expression of the *cyp13a3* gene (Figure 5). The expression of the *nhr-48* and the *cyp13a3* and *cyp1a1* genes was not correlated in most of the studied TBZ concentrations and times of exposure in L3 of *A. simplex* (s.l.).

## 3. Discussion

The mechanisms underlying anthelmintic resistance in parasitic nematodes are not fully understood, and there is a lack of sensitive diagnostics tools or methods to study the evolution of anthelmintic resistance or the impact of different control strategies [34]. Since translating the results of research conducted on *C. elegans*, i.e., free-living nematodes, into a parasitic organism is disputable and ambiguous, it is necessary to conduct a new analysis directly on the organism to which these studies relate to, or on a phylogenetically close model. An interesting hypothesis was that the regulatory elements of genes encoding xenobiotic response elements were less responsive to induction in the parasitic nematodes compared to the free-living nematodes [30], which suggested that response in the free-living *C. elegans* could not exactly be translated to parasites.

The albendazole effectiveness, a drug whose long-term use has induced resistance in many parasitic species [35,36], was tested on the *A. simplex* (s.l.) parasitic nematode directly. In vitro studies demonstrated that albendazole solubility increased in response to acidic pH, which inhibited glucose absorption through the nematode cuticle, thus increasing the parasite’s mortality [14,15]. This example suggests to us that the pathways by which anthelminthic drugs are metabolized and excreted in nematodes need to be characterized to broaden our understanding of drug efficacy and, most importantly, the relevant mechanisms of resistance. The TBZ effectiveness, a wide-spectrum anthelminthic with a higher efficacy than albendazole, has to date not been examined in *A. simplex* (s.l.).

The literature data suggest that, in parasitic nematodes, CYPs are involved in the phase I biotransformation of the antiparasitic drug. The CYP expression is regulated by NHRs, transcription factors that activate by binding specific ligands and that induce the expression of CYP encoding genes. The nuclear hormone receptors constitute an important superfamily of transcription regulators that are involved in widely diverse physiological functions, including the control of embryonic development and cell differentiation. In nematodes, NHRs play an important role during the development, molting, metabolism and differentiation of nerve cells. They can act as molecular regulators of homeostasis and gradient regulators. The receptors function as dimeric molecules in nuclei to regulate the transcription of target genes in a ligand-responsive manner. Nuclear hormone receptors consist of a highly conserved DNA-binding domain that recognizes specific sequences, connected via a linker region to a C-terminal ligand-binding domain. In addition, certain nuclear hormone receptors have an N-terminal modulatory domain. The ligand-binding domain acts in response to ligand binding, which causes a conformational change in the receptor to induce a response, thereby acting as a molecular switch to turn on the transcriptional activity [37,38]. Dimethylphenylpiperazinium, an anthelminthic drug, binds to the nuclear receptor DAF-12 (receptor of dafachronic acid) and inhibits L2/L3 molting and the development of Dauer larvae of *C. elegans* [39]. The metabolic processes in Dauer larvae and invasive (L3) larvae are similar [40], which suggests that TBZ might also act upon the nuclear receptor in *A. simplex* (s.l.). Additionally, according to Jones et al. [19], the TBZ could be one of those specific ligands.

Thus, during the treatment of *A. simplex* (s.l.) with TBZ, the effect of the *nhr-48* transcriptional modulator on the expression of the selected CYP genes was studied. This study demonstrated for the first time the presence of mRNA of NHR-48, a possible target receptor of TBZ, as well as the presence of mRNA of two CYP genes, *cyp13a3* or *cyp1a1,* in *A. simplex* (s.l.). The results of this study indicate that TBZ affects the expression of *nhr-48*, *cyp-13a3* and *cyp-1a1* genes in *A. simplex* (s.l.) (Figure 5). The correlation between the expression of *nhr-48* and *cyp13a3* (positive) or *cyp1a1* (negative) could point to the modulation of the expression of *cyp* isoforms by NHR-48 activation.

Literature data suggest that NHRs modulate the expression of cytochrome proteins and act as intermediaries between agonists and antagonists in drug biotransformation. In the cattle parasites *Cooperia oncophora* and *Ostertagia ostertagi*, the use of a CYP inhibitor (piperonyl butoxide) made the larvae of both strains more sensitive to anthelminthics, suggesting that CYPs play a role in metabolizing TBZ [41]. Menzel et al. [42] and Jones et al. [19] demonstrated that in *C. elegans* the metabolism of TBZ is controlled by *cyp*, whose expression is regulated by NHR. TBZ binds directly to NHR-176, which activates this transcription factor, enhances the expression of *cyp35d1* and induces the activation of the hydroxylation pathway [19].

The same families of *C. elegans* CYPs (1, 2 and 33) are orthologs of mammalian xenobiotic inducible genes, which are induced by ivermectin (IVM)—an anthelminthic from the macrocyclic lactones group [43,44]. Studies of *H. contortus* and *C. elegans* show that NHR-8 can regulate the transcription of genes associated with xenobiotic metabolism and transport and that the loss of *nhr-8* may lead to a decrease in IVM elimination, making the worms hypersensitive to the drug. Moreover, the inversely regulated gene expression of several genes encoding cytochromes in IVM-tolerant and *nhr-8* mutants further supports the hypothesis that IVM can induce the expression of genes involved in drug detoxification in an *nhr-8*-dependent manner [33]. While NHR-8 is known for the regulation of the xenobiotic metabolism [45], the NHR-48 annotated by our team in the *A. simplex* (s.l.) genome has not been studied to date regarding this possible function.

However, the results do not clearly confirm the possible correlation described earlier. It should be noted that the results are valuable from the point of view of research on NHRs and CYPs, much of which has so far been carried out on free-living nematodes rather than parasitic ones. Therefore, research on NHR-48 and CYPs in *A. simplex* (s.l.) should be continued and enriched with the results of LC-MS analyses of the xenobiotic metabolism as well as the inactivation/silencing of NHR-48.

## 4. Materials and Methods

### 4.1. Anisakis simplex (s.l.) Larvae

The experiment was performed on the third stage larvae of *A. simplex* (s.l.), which were isolated from Baltic herrings (*Clupea harengus membras*) purchased on the market in Olsztyn, Poland. The larvae were assessed under a stereo microscope as belonging to the *A. simplex* species type I [44,45]. Larvae were rinsed three times in a sterile saline solution (0.9% NaCl). Two hundred and ten larvae were then washed for 30 min in a bactericidal and fungicidal solution [80 mg of gentamicin sulphate (010807, PPH Galfarm, Kraków, Poland), 0.625 mg of amphotericin B (A9528, Sigma Aldrich, Poznań, Poland), 100 mg of chloramphenicol (107464, Pharma Cosmetic, Kraków, Poland), 10,000 IU of penicillin G (P3032, Sigma Aldrich, Poznań, Poland) and 4.5 mL of Hanks’ solution (H6648, Sigma Aldrich, Poznań, Poland) for a final volume of 10 mL of saline solution].

### 4.2. In Vitro Culture

All in vitro cultures were performed using RPMI-1640 medium (R8758, Sigma Aldrich, Poznan, Poland) enriched with 20% fetal bovine serum (F7524, Sigma Aldrich, Poznan, Poland) and 1% pepsin (P7125, Sigma Aldrich, Poznan, Poland) in a six-well plate (BD Biosciences) at pH = 4, 5% CO_2_ and 36 °C according to the method described by Iglesias et al. [46]. Larvae were cultured in vitro for 72 h with TBZ (dissolved in 0.1% dimethylsulphoxide (DMSO); 45684, Sigma Aldrich, Poznan, Poland) at five concentrations: 0.5 mM, 0.75 mM, 1.0 mM, 1.25 mM and 1.5 mM, based on Jones et al. [19]. The control culture did not contain TBZ (medium was with the addition of 0.1% DMSO). Larvae were examined under a stereoscopic microscope after 3 h, 6 h, 12 h, 24 h, 36 h, 48 h and 72 h to test the efficacy of TBZ. Larvae with no mobility or capture of the ventriculus and alteration of the cuticle were considered dead. After the culture, all L3 larvae were stabilized in StayRNA buffer (038-100, A & A Biotechnology, Gdynia, Poland) and stored at −80 °C for further analysis. The experiment was performed in triplicate.

### 4.3. Total RNA Isolation and cDNA Synthesis

The total RNA from L3 larvae was isolated with the Total RNA Mini Plus kit (036-100, A & A Biotechnology, Gdynia, Poland) according to the manufacturer’s instructions. cDNA was synthesized with 2 µg of RNA, oligo (dT) primers and reverse transcriptase from the TransScriba Kit (4000-100, A & A Biotechnology, Gdynia, Poland). The obtained cDNA was stored at −20 °C for further analysis.

### 4.4. Bioinformatical Analyses

Using the genomic data from our previous study [47], sequences encoding NHR and CYPs were taken for this analysis. Only *A. simplex* (s.l.) genes with the coding sequence previously annotated by Łopieńska–Biernat et al. [47] and deposited in the GenBank database (NCBI) were used in this study (*cyp13a3*, ID: KR092169; *cyp1a1*, ID: KX215154; *nhr-48*, ID: KR092170). Due to the lack of a precise characterization of the *cyp* and *nhr* genes in *A. simplex* (s.l.), it was decided to present it. Additionally, the protein structure of NHR was predicted. It was also decided to check the size of genetic changes between parasitic and free-living nematodes in the context of the studied genes.

Using Augustus [48] and Geneious Prime v. 11.0.6 + 10 tools [49], the intron-exon structure of *cyp1a1* and *cyp13a3* genes was characterized. Then, the translation products of CYP1A1 and CYP13A1 were used to search a larger pool of homologs in nematodes in two databases: the National Center for Biotechnology Information (https://www.ncbi.nlm.nih.gov/) and WormBase (https://wormbase.org//). For the final analysis, twenty-two protein sequences were taken from the family of CYPs identified in *Caenorhabditis elegans*, *C. briggsae*, *C. brenneri*, *C. remanei*, *Ascaris suum*, *Haemonchus contortus*, *Toxocara canis*, *Brugia malayi* and *Loa loa*. Multiple sequence alignment was performed in MAFFT [50] using the default parameters. The phylogenetic tree was built in Geneious Prime v. 11.0.6 + 10 [49] using Mr. Bayes plugin [51], based on the obtained alignment using the neighbor-joining clustering method.

The phylogenetic analysis, showing the origin and % of identity between *A. simplex* (s.l.) *nhr-48* and other nuclear receptors of different nematodes available in the National Center for Biotechnology Information (https://www.ncbi.nlm.nih.gov/) and WormBase (https://wormbase.org//) was built in Geneious Prime v. 11.0.6 + 10 [49] based on the obtained alignment (14 sequences) and using the method of neighbor-joining clustering and the PhyML plugin [52].

The 3D structure of the NHR-48 receptor was predicted using the SWISS MODEL tool [53] and protein model of *Ancylostoma ceylanicum* DAF-12 (nuclear receptor of dafachronic acid) (http://doi.org/10.2210/pdb3UP3/pdb) from Protein Data Bank [54] as a template with 55.6% identity and 33% coverage.

### 4.5. qReal-Time PCR

Real-time PCR was carried out using an ABI 7500 Fast Real-time PCR System (Applied Biosystems, Foster City, CA, USA) and an RT HS-PCR Mix SYBR B kit (2017-100B, A & A Biotechnology, Gdynia, Poland) according to the manufacturer’s instructions. The primers (cyp13a3, F: AATCGGTGCATTCGGAAGTG; R: TCCATCGTTCACCTCTTGCT; cyp1a1, F: AATGAAGAGCTGGACACCA; R: GAGTGCCTCATCAACCGTTC; nhr-48, F: TCATTGCTTCGATCAGTGCG, R: GAAGCTGTTGACGCCCATAG) used in the experiment were designed in Primer3 v. 0.4.0 [55]. The reaction conditions were as follows: template denaturation: 10 min at 95 °C, then 40 cycles of 15 s each at 95 °C, 60 s at 60 °C, and a final extension: 30 s at 72 °C. The specificity of the reactions was determined by analyzing the melting curves. Relative expression results were normalized with two Anisakis reference genes, elongation factor 1-alpha (*ef-1α*, GenBank KP326558; F: TCCTCAAGCGTTGTTATCTGTT; R: AGTTTTGCCACTAGCGGTTCC) and peptidyprolyl-cis-trans isomerase 12 (*ppi12*, GenBank KM496568; F: GGCACGATATTCCACAGGAT; R: CTCCATAGATCGATGCACCA) [56], to the control and analyzed by the 2^−ΔΔCt^ method [57].

### 4.6. Statistical Analysis

All in vitro cultures were performed in triplicate. The survival analysis was done using the Kaplan–Meier method. The median survival (%) was calculated. The survival curves were subjected to the Log-rank (Mentel–Cox) test (*p* ≤  0.05) and Log-rank test for trend (*p* ≤  0.05). The qReal-time PCR data were expressed as means ± standard deviation. An ordinary one-way ANOVA was performed for this data in the GraphPad Prism 8 software (GraphPad Software Inc., San Diego, CA, USA). Differences between the means were assessed by Dunnett’s multiple comparisons test. *p*-values below 0.1234 were considered nonsignificant, where 0.0332 (*), 0.0021 (**), 0.0002 (***) and <0.0001 (****) was significant. The Spearman correlation test between two datasets with the expression of a) *nhr-48* and b) *cyp1a1* or *cyp13a3* was performed to check if there was a correlation between the *nhr-48* expression and one of the CYP genes. The results were considered statistically significant when *p* ≤ 0.05 (one-tailed).

## 5. Conclusions

The TBZ efficacy against L3 larvae of *A. simplex* type I cultured in vitro was described for the first time. TBZ modified the expression of *nhr-48*, *cyp13a3* and *cyp1a1* genes in L3 of *A. simplex* (s.l.). In type II species (*A. physeteris*, *A. brevispiculata* and *A. paggiae*), we can expect differences in the expression level, but the overall trend should, in our opinion, be the same. Differences may result in the level of expression of the studied genes in relation to the diverse geographical distribution and definitive hosts of Anisakis spp., but in both types (I and II) the TBZ, from our assumption, would elicit a similar response in the context of the studied genes.

Further studies on the silencing of NHR-48, whose activation is possibly responsible for the biotransformation of TBZ in *A. simplex* (s.l.), might demonstrate that it could be an important target in studies on new antiparasitic drugs. It could also be hypothesized that *cyp13a3* and *cyp1a1* genes could be used during future studies focusing on the genes taking part in parasite-drug interactions related to drug resistance and new drug discoveries. Moreover, studies on xenobiotic metabolism pathways should be designed on the *A. simplex* (s.l.) model rather than on the *C. elegans* model (which is a free-living nematode) to increase the specificity of anthelmintic drugs.

## Supplementary Materials

The following are available online at https://www.mdpi.com/2076-0817/9/12/1030/s1, Supplementary File 1: The Genetic distance matrix of CYP sequences for different Nematode species. The values are expressed as a percentage. The higher the value, the higher the similarity. See Figure 3; Supplementary File 2: The Genetic distance matrix of NHR sequences for different Nematode species, and its graphical representation (phylogenetic tree of NHRs). The values are expressed as a percentage. The higher the value, the higher the similarity; Supplementary File 3: The results of the Spearman correlation test between two datasets of expression: (a) nhr-48 and (b) cyp1a1 or cyp13a3. Correlated conditions were highlighted in green. The results were considered statistically significant when *p* ≤ 0.05 (one-tailed).
